# COVID-19: Lessons Learned from Molecular and Clinical Research

**DOI:** 10.3390/ijms26020616

**Published:** 2025-01-13

**Authors:** Manuela Rizzi, Pier Paolo Sainaghi

**Affiliations:** 1 Department of Health Sciences, Università del Piemonte Orientale (UPO), 28100 Novara, Italy; 2 IRCAD (Interdisciplinary Research Center of Autoimmune Diseases), Università del Piemonte Orientale (UPO), 28100 Novara, Italy; 3 Department of Translational Medicine, Università del Piemonte Orientale (UPO), 28100 Novara, Italy

SARS-CoV-2 virus, the etiological agent of the novel coronavirus disease 19 (COVID-19), was first identified in late 2019, following the sudden appearance of a cluster of pneumonia cases of unknown origin in China. A few months later, the World Health Organization (WHO) declared the COVID-19 pandemic, and it has been recognized as the greatest and deadliest worldwide health emergency after the Spanish flu at the beginning of the last century. Even if the WHO Emergency Committee declared the end of the public health emergency of international concern (PHEIC) status in mid-2023, there are several areas of uncertainty concerning this disease and its pathogenesis, that remain to be elucidated [1,2].

Although in the last years wide efforts have been made in order to develop effective vaccines and targeted therapeutic approaches able to reduce the healthcare and socio-economic burdens of COVID-19, it is worth noting that the clinical features of the disease have changed, especially because of the high mutation frequency displayed by SARS-CoV-2 [3,4].

Since the beginning of the emergency, the first step to ensure effective COVID-19 management has been represented by the identification and quantification of the viral agent. The reference standard method for diagnosing the presence of SARS-CoV-2 has been represented by the real-time reverse transcription polymerase chain reaction (RT-PCR), a very accurate approach routinely used to detect viral RNA in biological samples. Unfortunately, especially during the first phases of the pandemic, many laboratories had to face an overwhelming demand for testing, highlighting the need for the development of new molecular tests able to quickly confirm the infection and guide clinical interventions, especially when immediate decisions about patient care are necessary. To fulfill this unmet need, several new technologies have been proposed, and some of them have been applied during the pandemic to screen with appreciable accuracy (mean positive detection rate = 78%, false-positive rate < 1% when used in the first week of symptom onset) large numbers of symptomatic cases, thus contributing to a more efficient and cost-effective use of the limited available resources [5,6]. Furthermore, in recent years, great efforts have been made worldwide to reduce COVID-19 spread through a global vaccination campaign. Considering that the immunity gained both through vaccination and past infections tends to wane over time, a quick detection of an ongoing infection still represents a great public health challenge. In this context, the availability of rapid and accurate antigenic tests represents a key issue for national and international policies intended to limit disease diffusion, especially nowadays, when, with the emergence of less lethal but more infectious viral variants, the number of asymptomatic or poorly symptomatic cases is increasing [7].

In relation to the lessons learned during the COVID-19 emergency, the development of accurate molecular tests alternative to RT-PCR will help to overcome its associated time-consuming nature, high labor costs, and possible reagent shortage, especially in resource-limited realities. Moreover, a wider range of diagnostic tools will also result in better management of future large infectious outbreaks and will also be helpful in assisting the following phase of transition toward long-term monitoring.

Once the infection has been detected, the successful management of patients who are infected relies on their early stratification based on the expected outcome in order to guide a prompt clinical intervention. Unfortunately, due to the novelty of the disease, no established and effective biomarkers were available at the beginning of the pandemic. For this reason, many researchers, through retrospective as well as prospective studies, tried to identify reliable markers able to drive clinical interventions [8,9,10].

These efforts in novel biomarker discovery led to the identification of several mediators, which have been introduced in current clinical practice as routine evaluations in the case of suspected and/or confirmed SARS-CoV-2 infection [11,12,13]. Moreover, due to the incessant search for increasing the specificity and sensitivity of diagnostic as well as prognostic biomarkers, many others have been identified and proved to be effective in specific cohorts of patients but have not yet been implemented in clinical practice [14,15,16].

The continuous improvements in this research field highlight the complexity of host responses elicited by SARS-CoV-2 infection, ranging from the activation of inflammatory pathways to antioxidant defense. According to these data, it appears of paramount importance to define specific biomarker panels able to support an early stratification of the patients who are infected according to the expected disease evolution and therapeutic response in order to optimize resource allocation and reduce disease-associated expenditure, especially in those realities where the availability of targeted therapies is limited.

During the COVID-19 emergency, health professionals also had to face the different prevalence of the disease among different age groups. As a matter of fact, for a long time it has been considered a disease that mainly affects adults, with the worst clinical presentation observed in the elderly and in high-risk subpopulations (i.e., comorbid and/or patients undergoing immunosuppressive therapy), while severe pediatric cases have been considered relatively uncommon [17,18,19,20]. Nevertheless, with the emergence of new variants, the scenario is changing, with an increasing number of confirmed infections also in the pediatric population, thus highlighting the need for a continuous monitoring of disease evolution also in this specific subpopulation, where SARS-CoV-2 infection could evolve towards a severe complication, represented by the so-called “multisystem inflammatory syndrome in children” (MIS-C) [21,22,23].

The COVID-19 pandemic, being the first global health emergency in the post-genomic era, had the opportunity to benefit from the innovative -omic technologies that have been developed in the last decades. In particular, these new technologies have been largely employed to sustain the understanding of viral infection origin, evolution, and spreading dynamics, as well as to support the development of novel diagnostic tools, targeted therapies, and vaccines. Considering that SARS-CoV-2, similar to other RNA viruses, tends to rapidly adapt to the changing environment by modifying its genome structure, resulting in the emergence of new variants with potential selective advantages, such as better immune escape and fitness in the final host, it is worth noting that genomic surveillance plays a critical role in monitoring disease evolution and in directing proactive measures to mitigate its global impact [24,25,26]. Genomic surveillance is based on the combination of epidemiological, genomic, and bioinformatic data to monitor the pathogen transmission and to predict its evolution towards new variants with altered pathogenic properties. At the beginning of the pandemic, the genomic surveillance network allowed the fast sharing of the novel pathogen genome sequence, thus supporting early identification of patients who were symptomatic, thus contributing to limiting viral spread by driving public health decisions. During the subsequent epidemic waves, instead, genomic sequencing has been of vital importance in the detection of the new emerging variants of concern and/or interest. To date, it is well recognized that its role is not limited to a passive monitoring of COVID-19 evolution, as it has been proven that constant surveillance is essential also for the development of diagnostic tools able to efficiently discriminate between viral strains and variants, for the implementation of public health and social interventions, and, finally, for the development of effective drugs and vaccines able to overcome SARS-CoV-2 immune escape ability [26,27]. According to that, the essential role of genomic surveillance in the management of infectious outbreaks emerges as another important lesson coming from the COVID-19 pandemic. Nevertheless, several challenges still require to be overcome, such as the economic burden of surveillance and the infrastructural disparities among high- and middle/low-income countries [28,29], thus fostering the development of new surveillance approaches based on more cost-effective technologies and integrating trainable machine learning algorithms, as well as the creation of large-scale consortia and integrated genomic surveillance networks, to support global preparedness toward the future pandemic emergencies.

The last few years have also been characterized by an increasing number of reports describing the development of chronic symptoms, lasting even for months after disease onset and finally resulting in a long COVID or post-acute COVID-19 syndrome diagnosis. This clinical condition is very heterogeneous, resembling a “chronic disease state” that could affect different clinical domains, in which the symptoms are different in terms of type and severity in each patient. Among the most common and representative long COVID manifestations are fatigue/muscle weakness; cognitive dysfunction; newly developed or exacerbated medical conditions, such as diabetes; cerebrovascular events; and autoimmune diseases, all conditions that can impair patients’ quality of life [30,31,32]. Epidemiological evaluations about this new syndrome are difficult, as its prevalence varies across regions, populations (patients who are vaccinated vs. unvaccinated), and SARS-CoV-2 variants. Although the lack of specific diagnostic tests or recognized biomarkers makes the correct diagnosis a clinical challenge, some risk factors have been identified, and among these, preexisting chronic conditions (especially cardiac, pulmonary, or renal) have been shown to play a role [30,32]. Apart from being a risk factor for developing long COVID, chronic diseases could also represent a sequelae of the primary infection [33]. Even if the pathobiology of long COVID is still not fully understood, dysregulated immune responses seem to play a critical role, accounting for the development of a self-sustaining proinflammatory condition leading to tissue damage and impaired regeneration [33,34]. Among the potential chronic sequelae of acute SARS-CoV-2 infection are the cardiovascular and hepatic ones, as suggested by the large number of patients experiencing cardiac symptoms or diagnosed with liver dysfunctions in long COVID cohorts. For these manifestations, a conceivable mechanistic explanation is represented by a chronic inflammatory response induced by viral reservoirs surviving primary infection resolution. As a matter of fact, both cardiac and hepatic tissues harbor ACE-2, which can act as a gateway for the virus and can lead to a dysregulation of the renin-angiotensin-aldosterone system homeostasis, finally resulting in tissue damage and organ function impairment (i.e., myocardial fibrosis, impaired perfusion and contractility, and potential arrhythmias or chronic liver complications such as steatosis, fibrosis, and cirrhosis) [35,36,37]. The existing evidence about the potential healthcare and economic burden associated with long COVID thus supports the need for targeted research and well-designed clinical trials aimed at implementing the management of this complex clinical condition that significantly affects patients’ quality of life.

Finally, even if extensive efforts have been made to develop targeted drugs for disease management during the pandemic, it is worth noting that, to date, only a limited number of specific and effective therapies are available on the market [38], thus fostering continuous efforts from the pharmaceutical industry in the development of new therapeutic agents able to effectively counteract SARS-CoV-2/host interactions. Considering that the most important limitation of the approved disease-specific therapies available is represented by the need to be administered in the early phase of the disease to be effective, it is not surprising the increased popularity of over-the-counter supplements that can act as therapeutic adjuvants or preventive agents toward SARS-CoV-2 infection, thus representing a cheap and easily accessible resource, especially in those clinical settings where resource availability is limited.

Considering this continuously evolving scenario, considerable efforts from the scientific community are needed to ensure a deeper understanding of COVID-19 pathogenesis, clinical evolution, and resolution. Thus, this Special Issue intended to collect the most recent advances in the field and, consequently, increase awareness for healthcare professionals regarding the challenges imposed by this disease, which has shown a great dynamic evolution, finally leading to the transition from emergency management to a new reality in which SARS-CoV-2 will be present alongside other endemic viruses.
